# F206. A TRANSLATIONAL STUDY OF BEHAVIOR, BRAIN STRUCTURE AND GENE PATHWAY IN ERBB4 KNOCKOUT MICE AND FIRST-EPISODE TREATMENT-NAïVE PATIENTS WITH SCHIZOPHRENIA

**DOI:** 10.1093/schbul/sby017.737

**Published:** 2018-04-01

**Authors:** Chengcheng Zhang, Peiyan Ni, Tao Li

**Affiliations:** 1 West China Hospital Sichuan University; 2 Mental Health Center, West China Hospital of Sichuan University

## Abstract

**Background:**

The current study was to explore how disruption of specific molecular circuits in the cerebral cortex may cause large-scale brain structure deficits and behavior changes via a translational study in conditional Erbb4 mutant mice and patients with schizophrenia.

**Methods:**

We conducted prepulse inhibition (PPI) and brain structural and diffusion magnetic resonance imaging (MRI) scans in 27 mice with ErbB4 knockout in parvalbumin (PV) interneurons and 23 age, sex-matched controls. Real-time quantitative polymerase chain reaction was used to assess the levels of five GABA-related transcripts in brain regions. We also measured structural and diffusion MRI and cumulative contribution of risk alleles in the GABA pathway genes using polygenic risk scores (PRS) in first-episode treatment-naïve schizophrenic patients (N=117) and age, sex-matched healthy controls (N=86).

**Results:**

ErbB4 knockout mice displayed behavioral deficit of PPI, as well as gray and white matter impairment in right sensorimotor cortical-striatal networks. We found significant correlations between gray matter volumes (GMVs) of the somatosensory cortex and PPI as well as GAD1 mRNA expression in controls but not in knockout mice. These findings were confirmed in a human sample where we observed significantly decreased gray and white matter impairment in sensorimotor cortical-striatal networks in schizophrenics. The PRS of GABA-pathway genes also displayed a negative correlation with the GMVs of the somatosensory cortex in patients.

**Discussion:**

Our study identified ErbB4 ablation induced prepulse inhibition deficits and GABAergic dysregulation in sensorimotor cortical-lateral striatal networks. We propose that ErbB4 signaling participates in sensorimotor gating dysfunction in schizophrenia by getting involved in somatosensory cortex deficits and GABAergic dysfunction.

